# There Is No Association between Coeliac Disease and Autoimmune Pancreatitis

**DOI:** 10.3390/nu10091157

**Published:** 2018-08-24

**Authors:** Giulia De Marchi, Giovanna Zanoni, Maria Cristina Conti Bellocchi, Elena Betti, Monica Brentegani, Paola Capelli, Valeria Zuliani, Luca Frulloni, Catherine Klersy, Rachele Ciccocioppo

**Affiliations:** 1 Gastroenterology Unit, Department of Medicine, AOUI Policlinico G.B. Rossi, University of Verona; Piazzale L.A. Scuro, 10, 37134 Verona, Italy; giuli.dema@yahoo.it (G.D.M.); mcristina.contibellocchi@gmail.com (M.C.C.B.); valeria.zuliani@univr.it (V.Z.); luca.frulloni@univr.it (L.F.); 2 Immunology Unit, Department of Pathology and Diagnostics, AOUI Policlinico G.B. Rossi, Piazzale L.A. Scuro, 10, 37134 Verona, Italy; giovanna.zanoni@aovr.veneto.it (G.Z.); monica.brentegani@aovr.veneto.it (M.B.); 3 Clinica Medica I, Department of Internal Medicine, IRCCS Policlinico San Matteo Foundation, Piazzale Golgi, 19, 27100 Pavia, Italy; elena.betti19@gmail.com; 4 Pathology Unit, Department of Pathology and Diagnostics, AOUI Policlinico G.B. Rossi, Piazzale L.A. Scuro, 10, 37134 Verona, Italy; paola.capelli@aovr.veneto.it; 5 Clinical Epidemiology & Biometry Unit, IRCCS Fondazione Policlinico San Matteo; Viale Golgi 19, 27100 Pavia, Italy; klersy@smatteo.pv.it

**Keywords:** autoimmune pancreatitis, coeliac disease, pancreatic disorders, screening

## Abstract

Autoimmune pancreatitis (AIP) is a rare disorder whose association with coeliac disease (CD) has never been investigated, although CD patients display a high prevalence of both endocrine and exocrine pancreatic affections. Therefore, we sought to evaluate the frequency of CD in patients with AIP and in further medical pancreatic disorders. The screening for CD was carried out through the detection of tissue transglutaminase (tTG) autoantibodies in sera of patients retrospectively enrolled and divided in four groups: AIP, chronic pancreatitis, chronic asymptomatic pancreatic hyperenzymemia (CAPH), and control subjects with functional dyspepsia. The search for anti-endomysium autoantibodies was performed in those cases with borderline or positive anti-tTG values. Duodenal biopsy was offered to all cases showing positive results. One patient out of 72 (1.4%) with AIP had already been diagnosed with CD and was following a gluten-free diet, while one case out of 71 (1.4%) with chronic pancreatitis and one out of 92 (1.1%) control subjects were diagnosed with de novo CD. No cases of CD were detected in the CAPH group. By contrast, a high prevalence of cases with ulcerative colitis was found in the AIP group (13.8%). Despite a mutual association between CD and several autoimmune disorders, our data do not support the serologic screening for CD in AIP. Further studies will clarify the usefulness of CD serologic screening in other pancreatic disorders.

## 1. Introduction

**Figure 1:**
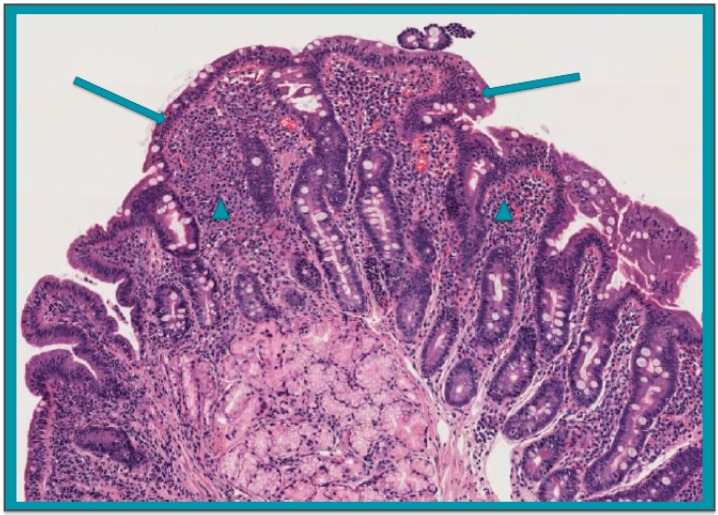
Histological features of duodenal mucosa of active coeliac disease showing subtotal villous atrophy with crypt hyperplasia and heavy lymphocytic inflammatory infiltrate in both the epithelial (arrows) and lamina propria (head arrows) compartments (hematoxylin-eosin, original magnification × 100).

Coeliac disease (CD) is an autoimmune condition affecting the small bowel mucosa of a proportion of subjects carrying the human leukocyte antigen (HLA)-DQ2 or -DQ8 haplotypes upon gluten ingestion [1]. Its prevalence, as assessed by serologic tests, is 0.4% in South America, 0.5% in Africa and North America, 0.6% in Asia, and 0.8% in Europe and Oceania, with higher values in female versus male individuals (0.6% vs. 0.4%; *p* < 0.001) [2]. The intestinal lesions encompass a variable degree of villous atrophy and crypt hyperplasia, with a heavy lymphocytic infiltrate of both the epithelial and lamina propria layers (Figure 1) [3]. The clinical picture is multifaceted, ranging from an overt malabsorption syndrome to apparently asymptomatic forms, with anaemia, isolated fatigue, cryptic hypertransaminasaemia, infertility, peripheral and central neurologic disorders, osteopenia, short stature, and dental enamel defects, being the main findings [1,4]. A gluten-free diet leads to an almost complete recovery of both mucosal lesions and clinical features in the vast majority of cases [1]. Remarkably, owing the same genetic and/or environmental predisposing factors, CD patients are at risk of developing further systemic or organ-specific immune-mediated disorders, with type 1 diabetes being the most prevalent and widely studied association, thus justifying the mutual serologic screening [5]. By contrast, no information about the possible association between CD and autoimmune pancreatitis (AIP), the immune-mediated condition affecting the exocrine component of the pancreas, is available so far.

**Figure 2:**
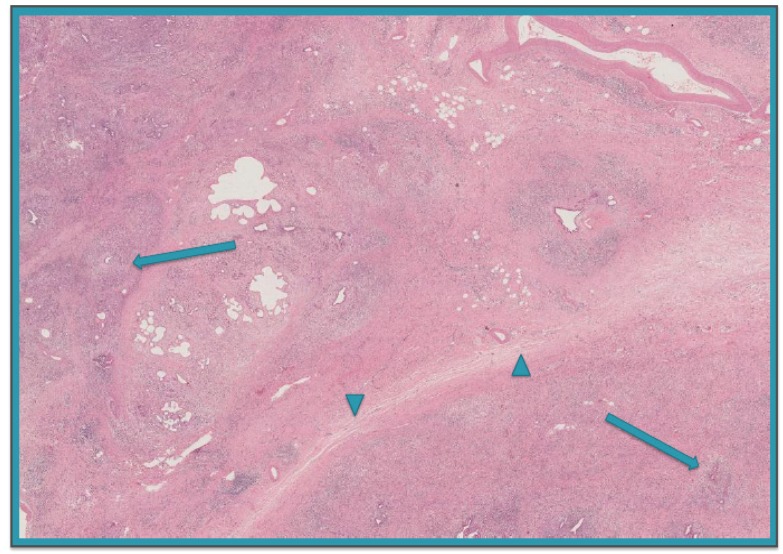
Histological features of autoimmune pancreatitis showing a dense periductal infiltration of plasma cells and lymphocytes leading to obliteration of the affected veins (**arrows**), and peculiar storiform fibrosis (**head arrows**) (hematoxylin-eosin, original magnification × 100).

AIP is a rare (estimated prevalence of 0.82:100,000 [6]), chronic fibro-inflammatory condition affecting the whole or a part of the gland, characterized by specific histological, radiological and serological aspects that disappear following a course of steroid therapy [7]. Two different types of AIP (type 1 and type 2) can be distinguished histologically. The first is the so called lymphoplasmacytic sclerosing pancreatitis displaying a dense periductal infiltration of plasma cells, mainly immunoglobulin (Ig)G4 positive, and lymphocytes, peculiar storiform fibrosis, and oblitering venulitis (Figure 2). The second, also called idiopathic duct-centric pancreatitis, is characterized by the presence of intraluminal and intraepithelial neutrophils in medium-sized and small ducts as well as in acini, often leading to destruction and obliteration of the duct lumen. However, the diagnosis of type 1 or type 2 AIP can be made even in the absence of histology by applying a combination of two or more of the following International Consensus Diagnostic Criteria [8]: (1) characteristic imaging features of both the parenchyma and main duct, i.e., a diffuse enlargement with delayed enhancement of the parenchyma with a long or multiple duct strictures without marked upstream dilatation in the typical form, while a segmental/focal enlargement with delayed enhancement of the parenchyma with segmental short duct narrowing in the atypical one; (2) increased level of IgG4; (3) other organ involvement, i.e., biliary duct, retroperitoneum, kidneys, salivary/lachrymal gland, as assessed histologically or radiologically; (4) response to steroid therapy. In those cases where distinctive criteria cannot be identified, the diagnosis of AIP not otherwise specified is given.

At variance with AIP, some evidence is available in the literature about the association between CD and non-immune-mediated disorders of the exocrine pancreas. Indeed, CD patients have been found to be at increased risk of developing both acute and chronic pancreatitis in comparison to the general population [9,10]. In addition, patients with villous atrophy, including CD, may carry an exocrine pancreatic insufficiency [11]. Finally, asymptomatic pancreatic hyperamylasemia, which usually precedes the diagnosis of CD and often disappears following a gluten-free diet, has been also described [12]. Similarly, macroamylasemia, a benign condition caused by circulating complexes of pancreatic or salivary amylases bound to plasma proteins that cannot be cleared by the renal glomeruli, has also been described in adulthood CD, but it possibly decreases or resolves after a strict gluten-free diet [13].

The aim of this study, therefore, was to establish the prevalence of CD in patients suffering from AIP by using the sera collected in the Biobank of a Tertiary Italian referral centre for pancreatic diseases. This gave us the unique opportunity to include also patients with non-immune-mediated pancreatic disorders, i.e., chronic pancreatitis and chronic asymptomatic pancreatic hyperenzymemia (CAPH), as control diseased groups, other than control subjects.

## 2. Patients and Methods

### 2.1. Study Population

Four groups of adult patients not taking steroids or immunosuppressive therapy at the time of blood sample harvest were enrolled in this study, as detailed below:

Group 1 (AIP). The sera of 72 out of 259 patients diagnosed with AIP (type 1 *n* = 43, type 2 *n* = 16, not otherwise specified *n* = 13) according to the International Consensus Diagnostic Criteria [8] were collected at the Pancreas Institute of the Policlinico G.B. Rossi (AOUI and University of Verona, Italy), from January 2003 through December 2017. Specifically, 40 out of 43 with type 1 AIP (93%), 2 out of 16 with type 2 AIP (12.5%), and 0 out of 13 with not otherwise specified AIP (0%) displayed IgG4 positivity.

Group 2 (chronic pancreatitis). A cohort of 71 out of 492 patients diagnosed with chronic pancreatitis from January 2012 to December 2017 was included in the study. The diagnostic criteria, as adapted following our experience, included at least one of the following criteria: (1) presence of pancreatic-type pain, history of acute/recurrent pancreatitis, presence of steatorrhea or diabetes, weight loss; (2) imaging findings of pancreatic parenchyma atrophy, main pancreatic duct dilation >6 mm and/or presence of irregularities, secondary ducts dilation, presence of pancreatic calcifications; (3) laboratory findings of decreased level of faecal elastase-1 (<100 μg/g of stool), glycated haemoglobin >6.5%; (4) histological features of chronic pancreatitis (loss of acinar cells, presence of interlobular fibrosis, infiltration of inflammatory cells and relative conservation of intralobular ducts and islets) in surgical specimens [14].

Group 3 (CAPH). This group comprised 32 out of 160 patients who were found with CAPH from January 2012 to December 2017. The diagnosis was made when the serum levels of lipases and/or pancreatic amylases were found above the upper normal limits (>10%) for at least three consecutive times lasting for more than six months in the absence of pancreatic-type pain [15]. Moreover, in all cases no lesions of the parenchyma and/or the ductal system were evident at the magnetic resonance of the abdomen with cholangiopancreatography sequences.

Group 4 (control subjects). The serum samples of a cohort of 92 patients suffering from functional dyspepsia, as assessed following the Rome III criteria [16], were collected from June 2012 to December 2016. The presence of relevant co-morbidities, such as primary immunodeficiencies, cancer, active infections or organ failure, was considered an exclusion criterion.

The demographic and clinical features of the study groups are listed in Table 1.

The Biobank of the Pancreas Institute, Policlinico G.B. Rossi, AOUI and University of Verona, Italy had been previously approved by the local Ethics Committee (Protocol number 5604, 2 February 2012). This study was approved by the local Ethics Committee (Protocol number 49061, 7 July 2018) and each enrolled patient gave written informed consent.

### 2.2. Screening for Coeliac Disease 

Detection of tissue transglutaminase (tTG) IgA antibody was performed by using a commercial Elisa test (Eu-tTG® IgA kit, Eurospital, Trieste, Italy; cut-off levels: negative < 9 U/mL, borderline 9–15 U/mL, positive > 15 U/mL). Patients with borderline or positive tTG IgA results underwent investigation for IgA anti-endomysium antibodies (EMA-IgA), which was performed by indirect immunofluorescent technique (Eurospital), according to the manufacturer’s instructions. Sera with low tTG IgA levels (<1 U/mL) were also evaluated by tTG IgG antibody determination (Eu-tTG® IgG kit, Eurospital, Trieste, Italy; cut-off levels: negative < 20 U/mL, positive ≥ 20 U/mL).

Duodenal mucosal sampling was offered to all cases with positive tTG-IgA and/or EMA-IgA. Four biopsies from the second part of the duodenum and two from the bulb were taken during upper endoscopy for the histological examination according to the Corazza–Villanacci classification [3].

### 2.3. Statistical Analysis

Continuous variables were expressed as the mean and standard deviation (SD). Discrete data were tabulated as numbers and percentages. The prevalence of CD was computed, together with its 95% exact binomial confidence intervals (95% CI) overall, for pancreatic disorders as a whole and by diagnostic group. Stata 15 (StataCorp, College Station, TX, USA) was used for computation.

## 3. Results

**Figure 3:**
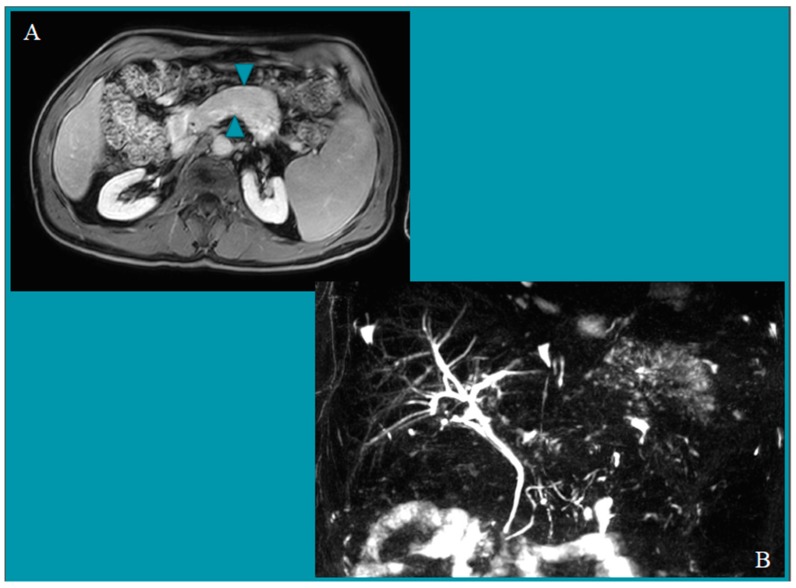
Abdominal magnetic resonance scan showing a diffuse enlargement of the body of the pancreas, with a “sausage-like” aspect (**A**), and multiple long stenosis of the main pancreatic duct at the cholangiopancreatography sequences (**B**).

A total of 267 serum samples harvested from 178 males and 89 females (mean age: 51.8 years, range: 18–85) were included in this study, with 175 being from patients with pancreatic disorders and 92 from control subjects with functional dyspepsia. Worth of note, a large prevalence of males was found in both AIP and chronic pancreatitis groups, accordingly with literature data [7,17], whereas a similar proportion of both genders was observed in the other two groups. As shown in Table 1, one case out of 72 patients of group 1 (1.4%), who was diagnosed with type 1 AIP, had already received the diagnosis of CD two years earlier because of unexplained hypertransaminasemia and weight loss. Since then, he was following a strict gluten-free diet with full recovery of laboratory and clinical features. Therefore, his CD serology resulted negative, and a normal mucosal architecture was found at histology (see Table 2). The radiological findings that led to the diagnosis of AIP type 1, together with a high level of IgG4, are shown in Figure 3. The serologic screening did not detect any further case of CD among patients affected by AIP. However, in two cases a search for tTG-IgG was carried out because of a very low level of tTG-IgA (less than 1.0 U/mL), giving negative results (4.34 and 6.25 U/mL). Remarkably, a consistent number of patients with AIP (13 out 72, 20.8%, of whom three had type 1 and 10 had type 2 AIP) was also affected by inflammatory bowel disease, mostly ulcerative colitis (10 out of 13 cases, while two had indeterminate colitis and one had Crohn’s disease). Specifically, one case was diagnosed with ulcerative colitis and AIP simultaneously, whereas the diagnosis of ulcerative colitis preceded that of AIP by a median interval of 28 months (range, 3 to 67 months) in the remaining patients. The vast majority of ulcerative colitis patients (eight out of 10) were not taking systemic corticosteroids at the time of diagnosis of AIP, although they had previously undergone this therapy; only a small proportion of them (three out of 10) was under biological agents (anti-tumour necrosis factor monoclonal antibody). Also, one patient amongst the 71 with chronic pancreatitis (1.4%) showed a positive value of both tTG-IgA antibodies, although at low titre (15,875 U/mL), and EMA-IgA at 1:5 dilution. The histologic examination of the duodenal biopsies showed the characteristic lesions, thus confirming the diagnosis of CD (see Table 2), and the patient was willing to start a gluten-free diet. When collecting his clinical history, aphthous stomatitis appeared evident. One further case was found within the group of patients with functional dyspepsia (1.1%), displaying positivity for both tTG-IgA (value 123 U/mL) and EMA-IgA at 1:16 dilution, thus leading to a definitive diagnosis of CD upon the demonstration of the characteristic lesions at histologic examination of the duodenal biopsies (see Table 2). Interestingly, she complained of infertility. An additional two cases in this group showed borderline values of tTG-IgA (12.44 and 13.47 U/mL) but was negative for the EMA test; hence, they did not undergo endoscopy, whereas in four cases a search for tTG-IgG was carried out because of a very low level of tTG-IgA (less than 1.0 U/mL), giving negative results (5.42, 5.73, 5.8, 8.15 U/mL). By contrast, no cases of positive CD serology were detected among the 32 patients with CAPH (mean value of tTG-IgA: 2.93 U/mL, range 1.276–7.537 U/mL). Therefore, as shown in Table 2, a total of three cases were identified to suffer from CD (two active and one treated) in the study population; that prevalence was similar in patients with pancreatic disorders and control subjects (overall prevalence 1.1%). Confidence intervals were consistent and ranged from 0% to about 10% in the single diagnostic groups and up to 4% in aggregated diagnoses.

## 4. Discussion

Limited information is available about the occurrence of exocrine pancreatic disorders during the course of CD [18], while strong evidence demonstrates an association between CD and type 1 diabetes [5,19]. In fact, approximately 90% of patients with type 1 diabetes carry either HLA-DQ2 or -DQ8 haplotypes as compared to 30% of the general population [20], with those diabetic cases homozygous for DR3-DQ2 having a 33% risk for the presence of tTG autoantibodies [21]. This is why the heterodimers encoded by these HLA haplotypes efficiently bind negatively charged peptides derived from gliadin upon tTG deamidation, thus eliciting a T- and B-cell mediated immune response [22]. This, in turn, leads to an upregulation of key pro-inflammatory molecules, such as interferon-γ [23] and interleukin (IL)-21 [24], responsible for tissue damage. It has also been suggested that dietary gluten could be involved in the pathogenesis of type 1 diabetes [25]. Conversely, a gluten-free diet largely prevented diabetes onset in non-obese diabetic mice, possibly through a modification of the gut microbiota [26].

However, autoimmune attack against the pancreas may involve not only the endocrine component, but also the exocrine one, giving rise to AIP. This is the pancreatic manifestation of the IgG4-related disease, whose genetic susceptibility and pathogenic mechanisms are still poorly understood [7]. Nonetheless, almost all of the candidate genes are directly or indirectly implicated in the regulation of the immune response [7]. A further aspect supporting an autoimmune background is the large proportion of AIP patients displaying autoantibodies, mostly against enzymes, such as lactoferrin, carbonic anhydrases, pancreatic secretory trypsin inhibitor, and trypsinogens [27,28]. Even CD is characterized by the presence of autoantibodies against a ubiquitous enzyme, tTG2, which, besides being the main autoantigen and target of the anti-endomysium autoantibodies [29], catalyses a specific and ordered deamidation of gliadin peptides, giving rise to immunodominant epitopes [22]. In addition, tTG2 seems to play a crucial role in the development of secondary autoimmunity through a post-translational modification of additional proteins, leading to the generation of neoantigens [30]. Also of note, transgenic HLA-DQ8 mice, grown in germ-free conditions and fed a gluten-free diet, developed acute pancreatitis after intra-peritoneal injection of cerulein, a cholecystokinin analogue that causes hyperstimulation of the exocrine component [31]. Whether or not gliadin was then introduced in the diet of these mice, an increased level of IgG1 (homologous of human IgG4) was observed, together with a histological pattern resembling that of human AIP [31]. Moreover, an increased level of serum IgG4 was documented in patients suffering from both CD and pancreatic exocrine insufficiency [32], while an increased number of IgG4+ cells was occasionally found at the mucosal levels of CD patients [33]. Finally, it is conceivable that the mucosal dysbiosis found in CD patients [34] might also contribute to an autoimmune attack in close organs, like the pancreas.

Despite these hypotheses, only one case suffering from both CD and AIP has been reported so far [35], thus we sought to investigate a putative association between these two immune-mediated conditions, taking advantage of the sera collected at the Biobank of a Tertiary Referral centre for pancreatic diseases. We found one patient among the 72 AIP patients who had already received the diagnosis of CD two years earlier. Therefore, the same prevalence of CD in AIP as that of the general population [2] was evident (1.4%). This also suggests that a gluten-free diet does not protect against the development of AIP. One possible explanation may lie in the different genetic predisposition since, at least in the Japanese population, an association of the DRB1*0405-DQB1*0401 haplotype with AIP was found [36], whereas CD is associated with the HLA-DQ2 and -DQ8 ones [1,5]. Moreover, unlike classic autoimmune diseases in which T-cells with regulatory effect are defective in number and/or function, they are likely activated in AIP. Indeed, an increased rate of transcription factor Forkhead box P3^+^CD4^+^CD25^+^ T-cells in both pancreatic tissue [37] and peripheral blood [38] was found in AIP patients, together with upregulation of two cytokines with modulatory functions, i.e., IL-10 and transforming growth factor-β [7]. These seem to be key molecules since the former contributes to IgG4 class switching [39], while the latter is involved in the development of fibrosis [40].

At variance with CD, a strong association between AIP and IBD (18.0% of cases), mostly ulcerative colitis (13.9%), has been found in our cohort of Caucasian patients, thus confirming previous reports [41,42]. However, the prevalence was higher than that found in either an American [41] or an Asiatic [42] study, where a frequency of 5.6% and 5.8%, respectively, was found. Likewise, both have a retrospective design and a similar sample size (71 and 104 AIP cases, respectively) [41,42]. However, the tools applied for the diagnosis of AIP were different, since the HISORt criteria for AIP [43] were used in the former, whereas the Asian Diagnostic Criteria for AIP [44] were used in the latter, thus possibly affecting the final results. The discrepancy may also be partly related to ethnic differences and to the relatively small number of patients recruited. Interestingly, the course of ulcerative colitis was worst in those suffering from both diseases since, during the follow-up period of 10 years, 33.3% of patients underwent a colectomy versus none of those suffering from ulcerative colitis alone [42]. Finally, although Berkson’s bias (patients with two uncommon diseases are more likely to be referred to a tertiary medical centre than patients with just one such disease) [45] could have inflated the magnitude of this association, our data strongly suggest that the AIP and ulcerative colitis are related to some degree whose extent deserves further investigation.

As far as the non-immune-mediated pancreatic disorders are concerned, it is widely acknowledged that both functional and anatomical changes of the gland may be caused by or coexist with CD [18]. A Swedish register study, indeed, found an increased risk of both acute and chronic pancreatitis in patients with adulthood CD during the observational period of 1964 to 2003 [9]. Furthermore, it was estimated that over 20% of patients with CD have defective exocrine pancreatic function [10]. This seems to be related to an impaired secretion of cholecystokinin pancreozymin secondary to enteropathy and/or malnutrition, since normalization of both intestinal mucosa and nutritional status restores the secretion of digestive hormones and enzymes [46]. Nevertheless, no information on the prevalence of CD in non-immune-mediated pancreatic disorders is available so far. We found one case in the chronic pancreatitis group (1.4%) and one in the control subject group (1.1%), again overlapping with the prevalence in the general population [2]. Thus, despite a relationship between CD and pancreatic disorders having been demonstrated, the opposite does not seem true. Accordingly, we did not find any positivity at the serologic screening for CD in the CAPH group, even though an abnormal elevation of serum amylase and/or lipase was found in CD patients but disappeared upon a course of gluten-free diet [12].

Obviously, our study has strengths and weaknesses. A point of strength is that this is the first study investigating the prevalence of CD in patients suffering from pancreatic disorders, whereas the studies published so far did the opposite. Moreover, if we consider that AIP is a rare and difficult-to-diagnose condition, the large sample size available, together with the appropriateness of the diagnosis, put us in a privileged situation where the putative higher prevalence of CD in this clinical setting might have been demonstrated, if there was any. The limitations include the retrospective design and the small sample size (and thus the relatively large confidence intervals) of the CAPH and chronic pancreatitis groups in comparison with the overall institutional cohorts due to the low level of willingness to give serum samples for future unknown studies, whereas the lack of sex and age matching among AIP and chronic pancreatitis patients with CAPH and control cases was largely expected [7,47]. In addition, serum IgG or IgG4 levels were not available in non-AIP groups because they are not routinely measured. Despite these limitations, our cohort of 72 patients with AIP represents one of the largest single-centre experiments to date.

## 5. Conclusions

In summary, our findings suggest that there is a low probability of there being an association of CD with AIP, thus serological screening for CD is not recommended in patients with AIP. By contrast, a strong association between AIP and ulcerative colitis appears evident. AIP is a relatively “new” diagnostic entity, thus further prospective and multicentre studies are needed to confirm the conclusions of this study.

## Figures and Tables

**Table 1:** Demographic and clinical features of the study population.

	Autoimmune Pancreatitis	Chronic Pancreatitis	Chronic Pancreatic Hyperenzymemia	Control Subjects
Number of cases	72	71	32	92
Male/female ratio	55/17	57/14	18/14	48/44
Mean age in years (SD)	56.5 (16.9)	55.1 (13.2)	52.7 (14.6)	45.7 (18.3)
Body mass index: kg/m^2^ (mean ± SD)	25.1 ± 4.1	23.2 ± 5.7	24.9 ± 4.4	22.7 ± 5.2
Time from diagnosis in months (mean ± SD)	25.4 ± 29.3	81 ± 37.5	n.a.	n.a.
Concomitant autoimmune disorders: IBD	13 (10 UC)	1 (Crohn)	0	1 (UC)
Thyroiditis	5	1	1	2
Psoriasis	3	0	0	1
Asthma	1	2	0	2
Coeliac disease	1 *	0	0	0
Rheumatic diseases	2	1	0	3
Thrombocytopenia	1	0	0	0

Abbreviation: SD: Standard Deviation; IBD: inflammatory bowel disease; n.a.: not applicable; UC: ulcerative colitis. * case already diagnosed with coeliac disease.

**Table 2:** Cases with positive results at the serological screening with their histological findings.

	N	tTG IgA	tTg IgG	EMA	Histology ^§^	Prevalence (95% CI)
Group 1	72	0	0	0	Grade A lesions *	1.4% (0.0–7.5)
Group 2	71	1	0	1	Grade B1 lesions	2.4% (0.0–7.6)
Group 3	32	0	0	0	Not performed	0% (0.0–10.9)
Group 4	92	1 + 2 borderline	0	1	Grade B2 lesions	1.1% (0.0–5.9)
Pancreatic disorders	175	1	0	1	−	1.1% (0.1–4.1)
Total	267	2	0	2	3	1.1% (0.2–3.2)

Abbreviations. EMA: anti-endomysium autoantibody; IgA: class A immunoglobulin; IgG: class G immunoglobulin; N: number of cases; tTG: tissue transglutaminase. ^§^ Following the Corazza–Villanacci classification [3]. * case already diagnosed with coeliac disease.

## Abbreviations

AIP	autoimmune pancreatitis
CD	coeliac disease
CAPH	chronic asymptomatic pancreatic hyperenzymemia
CI	confidence interval
EMA	anti-endomysium antibodies
HLA	human leukocyte antigen
Ig	immunoglobulin
SD	standard deviation
tTG	tissue transglutaminase
